# Insensibilité congénitale à la douleur

**DOI:** 10.11604/pamj.2014.18.197.4753

**Published:** 2014-07-05

**Authors:** Kawtar Inani, Fatimazahra Mernissi

**Affiliations:** 1 Service de Dermatologie, CHU Hassan II, Fès, Maroc

**Keywords:** Insensibilité congénitale à la douleur, neuropathies héréditaires, Congenital insensitivity to pain, hereditary neuropathies

## Image en médecine

L'insensibilité congénitale à la douleur (ICD), est une affection rare de transmission autosomique récessive, caractérisée par une absence congénitale de la perception de la douleur. Elle a été décrite pour la première fois en 1932 par DEARBORN, elle constitue le type V des neuropathies héréditaires sensitives et autonomiques (HSAN:hereditary sensory and autonomic neuropathies). Cette pathologie est caractérisée par l'absence de réaction à la douleur, l'absence d'anomalies neurologiques avec la présence d'automutilations. Le diagnostic différentiel se pose avec les autres types des HSAN. Plusieurs hypothèses ont été avancées pour expliquer la pathogénie de cette pathologie, dont la présence d'une mutation du Nav1.7 codée par le gène SCN9A situé sur le chromosome 2q24.3, responsable de l'incapacité à ressentir la douleur. Nous rapportons l'observation d'un adolescent de 15 ans, issu d'un mariage consanguin, unique de ses parents, et sans notion de cas similaire dans la famille, qui présente depuis l’âge de 4 mois une insensibilité à la douleur, la notion de mutilations post-traumatiques, ainsi qu'un retard psychomoteur. Et chez qui l'examen trouvait une absence des troisièmes phalangettes des deux mains, et des gros orteils, ainsi qu'un mal perforant plantaire au niveau du talent gauche. L'examen neurologique notait un respect de la sensibilité tactile, vibratoire, et thermale avec présence des reflexes tendineux. La prise en charge reposait sur des soins réguliers du mal perforant plantaire, ainsi qu'une sensibilisation de l'enfant et de sa famille sur les mesures d’éviction des traumatismes.

**Figure 1:**
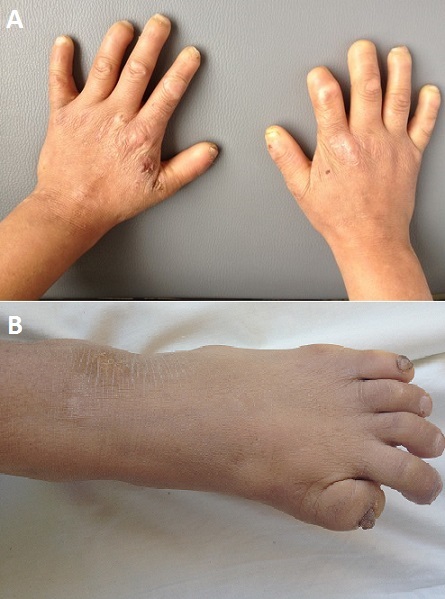
A) mutilation des dernières phalangettes; B) mutilation du gros orteil gauche

